# Preliminary Clinical Evaluation in Cross‐Sectional Imaging: A Qualitative Inquiry Into the Ethical Considerations in the Era of Artificial Intelligence

**DOI:** 10.1002/jmrs.70132

**Published:** 2026-10-14

**Authors:** Messiah N. K. Anudjo, Sharon Docherty, Theophilus N. Akudjedu

**Affiliations:** ^1^ Institute of Medical Imaging & Visualisation, School for Allied Health and Exercise Science, Faculty of Health, Environment and Medical Sciences Bournemouth University Bournemouth UK; ^2^ Department of Medical Technologies, School of Allied Health Professions and Pharmacy, Faculty of Medicine and Health Sciences Keele University Staffordshire UK; ^3^ Faculty of Health, Environment and Medical Sciences Bournemouth University Bournemouth UK

**Keywords:** artificial intelligence, cross‐sectional imaging, ethics, preliminary clinical evaluation, radiography

## Abstract

**Introduction:**

The evolving role of radiographers in Preliminary Clinical Evaluation (PCE), alongside the integration of artificial intelligence (AI) into radiography workflows, is reshaping professional boundaries and enhancing patient care. However, this introduces ethical challenges that may impact PCE performance. This study explored the perspectives of cross‐sectional imaging radiographers on the ethical dimensions of PCE and the potential impact of AI on the future practice of PCE.

**Method:**

A descriptive qualitative inquiry employing semi‐structured interviews was conducted. A Purposive sampling method was used to recruit twelve cross‐sectional imaging radiographers in a postgraduate medical imaging course at a UK university. Data were analysed using inductive reflexive thematic analysis with NVivo (v.20.0).

**Results:**

Participants' clinical experience ranged (2 to 8 years), with 50% (*n* = 6) employed in public hospitals and the rest in private or mixed settings. Half (*n* = 6) had expertise in both CT/MRI and the rest in CT only. Notably, 42% (*n* = 5) practised in multiple countries. Two main themes were developed: (1) Ethical considerations of PCE with subthemes surrounding privacy and confidentiality, communication boundaries, and legal compliance. (2) Prospects of human‐centred PCE in the era of AI, with subthemes including augmentation and support, workflow efficiency, PCE validation and quality assurance.

**Conclusion:**

Participants shared relevant ethical principles underpinning PCE practice and are cautiously optimistic about the potential impact of AI on PCE. Incorporation of PCE‐specific ethics into radiography education and practice is necessary to ensure consistency, accountability and safety. Cautious integration of AI into radiography practice workflows is recommended to avoid professional deskilling and to preserve expertise.

## Graphical abstract

This study explored the perspectives of cross‐sectional imaging radiographers on the ethical dimensions of PCE, and the potential impact of AI on its future practice through a qualitative lens. Core ethical considerations include privacy, confidentiality, communication boundaries, and compliance with legal frameworks. While AI integration offers opportunities for augmentation, workflow optimisation, and quality assurance, its adoption must be systematic and ethically grounded to prevent professional deskilling and preserve radiographer expertise and patient‐centred care.

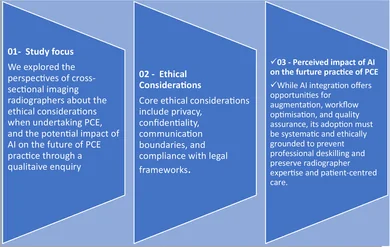

## 1 Introduction

Historically, image interpretation has been the reserve of radiologists while radiographers focus primarily on image acquisition [1]. Although radiographers in jurisdictions such as the United Kingdom (UK) and Australia have long participated in some form of image evaluation, this was primarily limited to flagging perceived image abnormalities through the ‘red dot system’ in projectional radiography within emergency settings [2, 3]. This model of practice is evolving, with radiographers now expected in many jurisdictions to undertake preliminary clinical evaluation (PCE)/preliminary image evaluation (PIE) on images with significant or time‐sensitive abnormalities in both projectional radiography and cross‐sectional images, including computed tomography (CT) and magnetic resonance imaging (MRI) [4].

These practice changes have been driven by rising imaging demands, a global shortage of radiologists, technological advances, and the increasing integration of artificial intelligence (AI) into clinical radiology; factors that are reshaping professional boundaries within clinical radiology [5, 6, 7, 8].

PCE/PIE is a radiographer‐led image evaluation and commenting process primarily intended to support ongoing patient care [9], particularly in emergencies where a radiological report may not be readily available. It involves radiographers assessing medical images, making informed judgements, and communicating their findings clearly and in writing to the referrer [10]. In contrast, radiographer reporting is an advanced practice that provides a definitive interpretation report on medical images by radiographers who have completed an accredited postgraduate training in a specific area of clinical image reporting [9, 10].

These practices support timely diagnostic decision‐making and contribute to effective patient management [1, 9]. In particular, the importance of PCE may be particularly evident where early recognition and escalation of life‐threatening pathology such as intracranial haemorrhage may support urgent and potentially life‐saving interventions, particularly when a definitive radiological report is not immediately available.

Within the UK context, the Society and College of Radiographers (SCoR) encourages transition from abnormality‐signalling systems, such as the red dot, to structured written PCE but cautions that implementation or extension should be in accordance with healthcare organisations' local circumstances [10]. In cross‐sectional imaging, however, the SCoR emphasises the recognition and escalation of clinically urgent findings through established local escalation procedures.

Moreover, the UK's Health and Care Professions Council (HCPC) standards of proficiency [11] require diagnostic radiographers to be competent at differentiating normal and abnormal image appearances and communicate these appropriately. The HCPC further requires radiographers to escalate urgent or unexpected findings in any imaging modality when no PCE system is in place, as outlined in standards 13.7 and 13.40. Similarly, the Medical Radiation Practice Board of Australia (MRPBA) minimum competency framework [12] requires that all radiographers are able to identify significant pathology and communicate their findings to the referrer to ensure patient safety.

PCE/PIE offers significant potential to leverage radiographers' expertise in the prompt identification of abnormal imaging findings [9]. Reported benefits of PCE include reductions in error rates, misdiagnosis, and patient recalls [13, 14] as well as positive effects on patient morbidity and mortality [15, 16].

Despite the benefits of PCE, formalisation, compliance, and consistent application of practice guidance where available remain a challenge. Tonk and colleagues (2023) [17], for example, reported that although professional bodies in Australia endorse written PCE, practice remains informal, inconsistent, and often undocumented. A UK study [18] in 2023 highlighted similar issues and identified gaps, including a lack of specificity, with implementation appearing somewhat regional and not universally employed. Similar findings were reported in a recent international study [4] focusing on cross‐sectional imaging radiographers, which highlighted a lack of practice guidance and inconsistencies in practice implementation as key barriers to wider adoption of PCE.

While PCE has traditionally been associated with projectional radiography, its expansion into cross‐sectional imaging may introduce distinct ethical challenges. Unlike projectional radiography, CT and MRI image evaluation involves more complex anatomical and clinical reasoning and frequently reveals time‐sensitive, incidental, or unexpected findings that may require urgent communication or escalation. Radiographers undertaking PCE in these modalities may therefore assume greater responsibility for recognising, prioritising, and communicating clinically significant abnormalities while navigating professional scope‐of‐practice boundaries and accountability requirements. Decisions regarding the escalation of incidental findings may also present ethical dilemmas less commonly encountered in projectional radiography. Consequently, ethical considerations identified within the projectional radiography literature, such as those described by Smith (2006) [19], may not be directly transferable to cross‐sectional imaging practice.

Despite the growing adoption of PCE in cross‐sectional imaging, little is known about how radiographers perceive and navigate the ethical responsibilities associated with this practice. Recent scoping reviews examining the state and scope of PCE internationally [1, 20] indicate that existing research has focused predominantly on education, diagnostic performance, implementation, and workforce issues, with limited attention given to ethical considerations. Although guidance on the management of incidental findings exists in research settings [21] such recommendations may not be directly transferable to clinical practice because of differences in professional roles, responsibilities, and scope of practice. Consequently, important questions remain regarding professional accountability, role boundaries, and the communication and escalation of clinically significant findings. This evidence gap is particularly significant given the inconsistencies in PCE implementation and the absence of internationally recognised frameworks governing practice [22]. As radiographers assume increasing responsibility for the evaluation and escalation of CT and MRI findings, a clearer understanding of the ethical challenges underpinning PCE is required. Thus, knowledge of, and adherence to, ethical principles are essential to ensure patient safety and consistency in service delivery [23]. While key publicly available professional PCE practice guidelines [10, 11, 12] clearly set out expectations and scope of radiographer PCE, ethical norms or considerations specific to PCE were not explicitly stated. Coupled with the potential legal implications of PCE practice, this highlights a need for a context‐specific understanding of the ethical considerations underpinning PCE. Such understanding may help safeguard both radiographers and patients. This may also shape the development of PCE curricula by reinforcing key PCE‐specific ethical principles that radiographers must be taught during their training.

In addition to these ethical considerations, the increasing integration of AI into medical imaging introduces further questions regarding responsibility, professional judgement, and decision‐making in PCE practice.

AI is increasingly influencing radiography practice, particularly in medical imaging and radiotherapy [24]. Although radiographers have historically adapted to technological advancements in medical imaging, AI introduces unique opportunities and challenges. Empirical evidence has shown that AI is already transforming radiology workflows, enhancing the efficiency of radiographic practice, supporting task prioritisation, improving diagnostic confidence, and informing treatment strategies [25, 26, 27, 28]. A global survey by Akudjedu and colleagues [25] on radiographers' knowledge, perceptions, and expectations of AI highlighted these benefits, but also identified notable concerns, including potential de‐skilling and reduced patient‐centred care due to over‐reliance on advanced technologies. Similarly, a large European survey conducted by Stogiannos and colleagues [24] which involved over two thousand European radiographers from 37 countries regarding the perceived impact of AI on professional identity, careers, and radiographers' roles further echoed some of the findings previously reported by Akudjedu et al. [25]. Notwithstanding, generally radiographers viewed AI as a tool to augment, rather than replace, their expertise in radiography practice. In the context of image interpretation, a study by Rainey and colleagues [29], on the perceptions of reporting radiographers in the UK found that they would be more confident of their diagnosis if they had agreement from an AI tool and that disagreement would drive them to seek a second opinion. Thus, highlighting an overall positive attitude towards the integration and applications of AI into clinical practice. For example, AI models are enabling radiographers to engage in advanced image analysis, thus blurring traditional radiological role boundaries and expanding their scope of practice [30, 31].

Though radiographers generally perceive AI as having a potentially positive impact on various aspects of radiographic practice and professional roles, its influence on the emerging responsibility of radiographers in the provision of PCE in the context of cross‐sectional imaging remains unclear. This ambiguity highlights the need for a more targeted exploration of radiographers' perceptions regarding how AI may affect their role and engagement in PCE. The UK SCoR's updated PCE guidance [9] recognises the potential of AI to support abnormality detection and clinical decision‐making; however, it cautions that recommendations for routine use should be based on a demonstrable evidence base, as current AI systems have not yet undergone full technology evaluation.

This study aimed to explore cross‐sectional imaging radiographers' views on the ethical responsibilities associated with PCE and their perceptions of how AI integration may influence the future sustainability of the radiographer's role in PCE delivery.

The findings of this study form part of an already published qualitative study by the authors exploring the perspectives and lived experiences of internationally trained cross‐sectional imaging radiographers regarding PCE education, training and practice [4].

## 2 Method

### 2.1 Ethical Considerations

This research received institutional ethics approval from Bournemouth University's Research Ethics panel. Data collected were securely stored in the lead author's institutional Microsoft OneDrive. Finally, all participants were informed that their participation was entirely voluntary, and they were at liberty to withdraw from the study at any time without giving a reason.

### 2.2 Study Design and Setting

The study employed qualitative research methodology [32] underpinned by a qualitative descriptive (QD) design [33]. QD methodology is particularly suited to studies seeking a rich, straightforward account of participants' experiences and perceptions while remaining close to the data and participants' own language [33, 34].

Unlike other qualitative designs such as phenomenology (which seeks to uncover the essence of lived experiences), grounded theory (concerned with the generation of theories) and ethnography (which focuses on understanding cultural practices and social groups), the QD approach focuses on providing a comprehensive summary of participants' experiences without deep theorisation or re‐contextualisation [35]. Grounded in naturalistic inquiry, QD facilitates the study of phenomena in their natural context and enables researchers to present a direct description of participants' perceptions and experiences [34]. Furthermore, the design offers methodological flexibility through the use of diverse sampling strategies, theoretical perspectives, and data collection methods [36].

Given that this study sought to explore cross‐sectional imaging radiographers' ethical considerations when undertaking PCE, and their perceptions of the future of PCE in this era of widespread integration of AI into medical imaging workflows without the intention of uncovering lived experiences or generating theories, the QD methodology was considered an appropriate approach for obtaining detailed accounts of participants' views and experiences.

The research participants were cross‐sectional imaging radiographers enrolled in a postgraduate imaging programme at a UK higher education institution. All but one of the participants had obtained their initial radiography qualification outside the UK and had prior clinical experience in their home countries, while most were also actively practising in the UK at the time of the study. The diverse educational, cultural, and healthcare practice backgrounds of these participants were considered valuable for the study's exploratory aim of examining the topic from a broader international perspective. Although radiographers recruited directly from UK clinical settings could also have provided useful insights, their experiences may have reflected more specific local contexts rather than the international perspective the study sought. Furthermore, to the best of our knowledge, no previous study has examined this topic within either the cross‐sectional imaging domain or an international context. Consequently, these participants were considered well positioned to provide exploratory insights to inform future research in this area.

### 2.3 Reflexivity and Researcher's Positionality

Reflexivity is a core principle in qualitative research and involves the researcher critically examining how their own background, beliefs, experiences, values, and assumptions may influence the research process and the production of knowledge [37]. Reflexivity acknowledges that research is not a neutral activity; rather, researchers actively contribute to the construction and interpretation of data.

The authors of this study are researchers and academics with varying levels of experience. The principal author (MA) is an early‐career mixed‐methods researcher, medical imaging educator and qualified diagnostic radiographer with extensive clinical experience in cross‐sectional imaging (MRI and CT), practising in both low‐resourced and high‐resourced contexts. MA is also a graduate of the postgraduate medical imaging programme from which the research participants were recruited. Although this created a degree of familiarity with the training context, MA had no prior personal relationship with any of the research participants, nor had he known any of the participants in person. His clinical and educational expertise provided deep contextual understanding of the phenomenon; however, he maintained an open and sensitive stance throughout by being reflective and critical towards the data and not imposing pre‐understanding and assumptions of the phenomenon on the data [38]. The co‐authors (TNA and SD) served as a check to ensure that assumptions about expected competencies or teaching practices during the interviews and analysis were not imposed. This was particularly facilitated by having the second author (SD) in the team, who is not a radiographer but a seasoned researcher.

The second author (SD) has over two decades' experience conducting health research and teaching research methods at all levels of higher education. The third author (TNA) is an associate professor in clinical imaging, a qualified diagnostic radiographer and an experienced academic and researcher in radiography. Both SD and TNA hold PhDs in their respective fields and provided methodological oversight throughout the study. Their research experience and academic status offered an important distance from the clinical setting, which has helped to challenge the data analysis and support reflexive rigour.

All the interviews were conducted by MA with support from SD and TNA. Prior to the data collection, MA completed formal training in qualitative research, including interviewing, recruitment, and thematic analysis. At the beginning of each interview, the interviewer introduced himself, clarified the purpose of the study, and obtained verbal confirmation of consent. Reflexive notes were kept after each interview to document impressions, assumptions, and potential influences on the data. Analytical decisions were discussed among all authors to ensure transparency and enhance trustworthiness. Even though formal reflexivity is not required in a qualitative descriptive study, given the lead author's professional and academic backgrounds, reflexivity was employed to ensure that their assumptions do not influence the data collection and analysis and that results interpretation remained closely aligned with the research participants' accounts.

Of note, given potential power imbalances between the researcher and the students, participants were informed that participation was entirely voluntary and were given sufficient time to independently decide their participation in the study. Additionally, potential coercion was minimised by informing participants that their participation in the study is independent of their educational or supervisory relationships and would not influence their academic progress or assessment outcomes.

### 2.4 Sampling and Data Collection

Twelve cross‐sectional imaging radiographers from the student cohort participated in this study. Purposive sampling [39] was employed to recruit the participants through their HEI in September 2024. A 10‐min online presentation via Microsoft Teams was made to the entire student cohort from which the participants were recruited. This was necessary to explain the aim of the research and to extend an invitation for participation. Eligible participants who expressed interest and shared their email address were contacted through their student email to arrange the interview.

In‐depth, semi‐structured interviews were conducted from December 2024 to February 2025 using an interview guide informed by a previous scoping review [1]. The guide was developed by the lead author (MA) with input from an expert clinical academic research cross‐sectional imaging radiographer with cross‐cultural clinical experience. It was further reviewed and refined by two experienced researchers (SD and TNA) to ensure validity and consistency of questioning across the research participants while allowing flexibility. The peer review by SD and TNA also ensured that the questions were truly open‐ended, non‐leading, and aligned with the research's aim.

All interviews were carried out, audio recorded and transcribed through Microsoft Teams (v 25185.410.3812.8024) with prior consent and permission of the interviewee. The lead author (MA) conducted all the interviews. Each interview lasted a maximum of 30 min. By the seventh interview, no new theoretical insights emerged; however, the rest of the interviews were completed as reflexive thematic analysis [40] does not support the concept of saturation. This also ensured any potential outlier perspectives were captured and reinforced the consistency of findings. To ensure accuracy and transparency of the data, participants were given the opportunity to review their individual interview transcripts. Of note, participants were interviewed individually to ensure confidentiality and to protect their privacy.

### 2.5 Data Processing and Analysis

Data cleaning was carried out to correct grammar and spelling errors in the individual transcripts. Each transcript was pseudo‐anonymised [41] by removing personally identifiable information and assigning participant codes as follows: P1, P2, P3, P4, P5, P6, P7, P8, P9, P10, P11, and P12.

### 2.6 Data Analysis

Data analysis and reporting followed Braun and Clarke's six stage reflexive thematic analysis approach (Figure 1) [40]. NVivo software (v20.0) was used for organising and coding the data while providing an audit trail. An inductive coding approach was used to identify significant and relevant phrases allowing the data to be interpreted directly from participants' expressions to support a credible and reliable analysis. The inductive coding approach was preferred as it captures meanings directly from what participants have said, driven by the data, thereby giving voice to participants' narratives of their experiences. Themes were then developed from the codes through an iterative process of multiple reviews and refinement. Bracketing [42] was carried out to ensure potential researcher biases were set aside during the analysis while promoting transparency, trustworthiness and credibility of the findings. This was supported through multiple consensus meetings by the research team (MA, TNA and SD) to review the transcriptions, codes and the overall results emanating from the thematic analysis.

**FIGURE 1:**
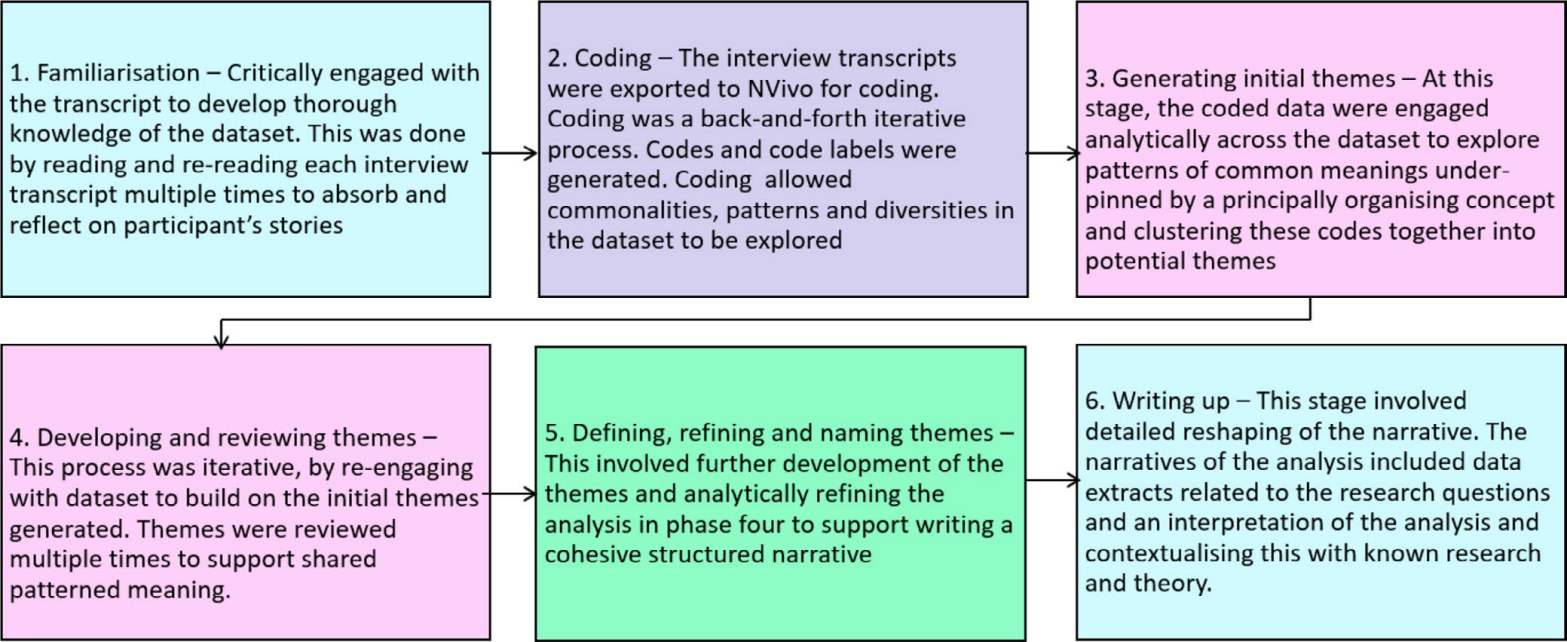
Braun and Clarkes six phase reflexive thematic analysis [40].

## 3 Results

### 3.1 Demographic Characteristics

A total of twelve cross‐sectional imaging radiographers participated in the study, comprising six CT radiographers and six with expertise in both MRI and CT. Participants' clinical experience ranged from 2 to 8 years, with the majority (*n* = 10, 83%) having less than five years' experience. Employment sectors varied, with half (*n* = 6) working in public hospitals, while the remaining participants were employed in private hospitals or held positions in both public and private sectors. Regarding international experience, 42% (*n* = 5) of the participants had practised in more than one country (Table 1). Additional demographic information is available in Part One of the study [4] previously published by the authors.

**TABLE 1:** Participants' demographic characteristics.

Demographic characteristics	Results
Work experience	
2–5 years	10 (83%)
≥ 6 years	2 (17%)
Sector of employment	
Public hospital	6 (50%)
Private hospital	3 (25%)
Public & private hospital	3 (25%)
International practice	
Single country	7 (58%)
Multiple countries	5 (42%)
Area of clinical expertise	
CT	6 (50%)
CT & MRI	6 (50%)

The thematic analysis yielded two main themes and six subthemes related to the ethics of PCE and the impact of artificial intelligence on PCE (Figure 2).

**FIGURE 2:**
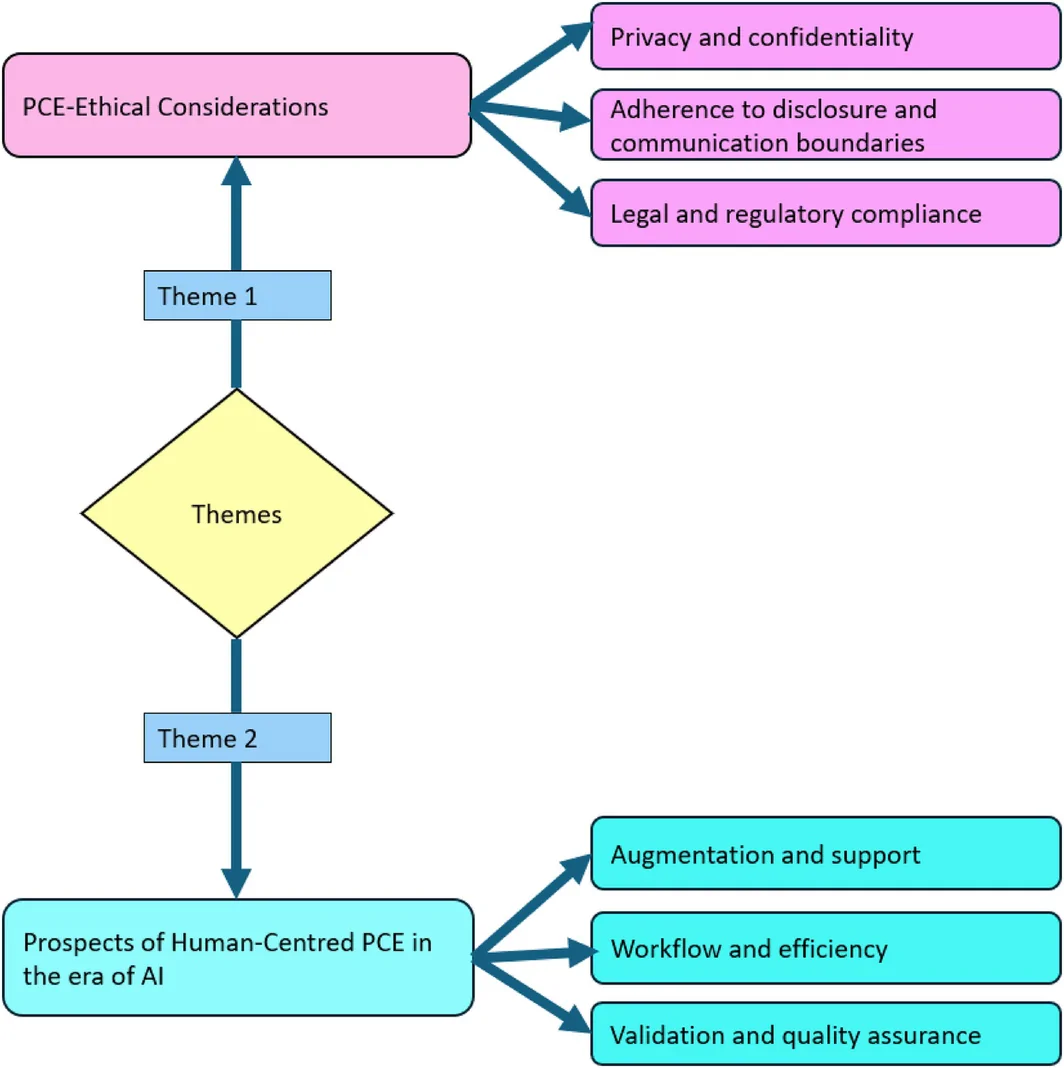
Themes and subthemes in relation to ethical considerations and AI in the practice of PCE.

### 3.2 Theme 1: PCE‐Ethical Considerations

Participant's demonstrated strong awareness of the pertinent ethics associated with the performance of preliminary clinical evaluation. Key ethical concerns identified included maintaining professional boundaries when disclosing imaging findings, safeguarding patient confidentiality, and ensuring compliance with legal and institutional regulations and protocols. These ethical values are considered crucial to ensuring safe and responsible preliminary clinical evaluation.

#### 3.2.1 Subtheme: 1.1: Privacy and Confidentiality

Keeping diagnostic imaging findings confidential emerged as a central obligation when performing PCE. Participants emphasised that any impression formed during the performance of PCE should be withheld from any unauthorised people. Participants underscored the importance of safeguarding patients' information and ensuring that their PCE comments remain confidential.

*P2* ‘Considering ethical practices and patient confidentiality is crucial.’ *(MRI and CT Radiographer with experience working in Ghana)*.

*P5* ‘I'd say that patient's privacy and consent are very important examples in PCE practice.’ *(CT and Radiographer with experience practicing in Kenya and the UK)*.

*P3* ‘We must maintain patient privacy…’ *(MRI and CT Radiographer with experience working in Ghana and UK)*.

#### 3.2.2 Theme 1.2: Adherence to Disclosure and Communication Boundaries

Participants in this study consistently acknowledged that radiographers are not permitted to discuss the findings from their PCE with patients. Participants' responses underscored the need for radiographers to be aware of their ethical obligation of following appropriate communication channels. As necessary, PCE findings should only be communicated solely to referrer clinicians or the radiologist. These responses also highlight the participants' understanding of their professional boundaries in the diagnostic process. However, the responses did not suggest any variations in the ethical obligations in this context. There seemed to be consensus on the channel of disclosure of PCE findings regardless of where participants were trained or practiced.

*P3* ‘Radiographers are not allowed to share any information about the diagnosis… We really don't disclose anything to the patient. So, we just tell them to go back to their referring doctor. So basically, using the right channel of information and communication of what we see to the referrer.’ *(MRI and CT Radiographer with experience practising in Ghana and UK)*.

*P11* ‘Radiographers should not discuss imaging findings with patients. Findings should be shared with the referring clinician only’ *(MRI and CT Radiographer with experience practicing in Pakistan)*.

#### 3.2.3 Theme 1.3: Legal and Regulatory Compliance

Participants identified adherence to national and institutional regulations or protocols as important ethical practice when undertaking PCE. They acknowledged that ethical practice is tied to compliance with hospital policies and professional standards pertaining to PCE. Thus, availability of detailed and comprehensive protocols; local or national guidance that regulates PCE practice is considered crucial. Compliance with these regulations or protocols can assure the safety of both patients and radiographers.

*P8* ‘In the UK, we use guidelines for PCE, but I find them lacking in detail. …a detailed protocol or SOP for radiographers would be helpful. Also, better system for conveying PCE information would help. We have a red dotting system in general radiography, but not for CT scans.’ *(Specialist CT radiographer with experience practicing in the UK and New Zealand)*.

*P12* ‘We must adhere to local rules and regulations, including those set by the Care Quality Commission and hospital policies. Ethically, as radiographers, we have limitations. We should not discuss or disclose any findings to the patient, as this is the responsibility of the consultant physician. Discussing imaging findings with patients can lead to contradictions if our interpretation differs from the radiologist's report.’ *(MRI and CT Radiographer with experience practicing in Pakistan and UK)*.

### 3.3 Theme 2: Prospects of Human‐Centred PCE in the Era of AI

Participants acknowledged the growing influence of AI within radiographic practice, particularly in relation to image interpretation. While they recognised AI's potential to enhance the efficiency and accuracy of PCE, they emphasised that it should function only as a supportive tool rather than replace radiographers' expertise. Overall, participants did not perceive AI integration as a threat to PCE; instead, they viewed it as a complementary resource that can assist radiographers in delivering high‐quality patient care.

#### 3.3.1 Theme 2.1: Augmentation and Support

Radiographers who participated in this study viewed AI as a valuable adjunct that can enhance, but not replace, their role in image interpretation. They advocated for a collaborative approach, where AI supports radiographers in delivering high‐quality PCE. Participants further highlighted that AI is not yet universally adopted, especially in certain clinical settings and that successful integration requires proper training, ethical safeguards, and clear frameworks.

*P4* ‘I believe radiographers will continue to be vital in the image interpretation process, especially in situations where technology has yet to become widespread or is in the early stages of adoption.’ *(CT Radiographer, with experience practicing in Rwanda)*.

*P5* ‘I think that AI and technological advancements will benefit image interpretation, but AI cannot replace radiographers' essential role in providing initial comments/PCE on medical images, especially since AI algorithms are still being trained to be more effective and accurate.’ *(MRI and CT Radiographer with experience practising in Kenya and UK)*.

#### 3.3.2 Theme 2.2: Workflow and Efficiency

Participants in this study highlighted AI's potential to streamline medical imaging workflows, aid workload reduction, and standardise PCE. However, they also stressed the need for proper training and ethical safeguards to ensure responsible use while keeping radiographers engaged and their expertise central to PCE practice.

*P2* ‘AI can aid in workflow management and image interpretation, but proper frameworks and training are essential to ensure safe and ethical use of patient information.’ *(MRI and CT Radiographer with experience practising in Ghana)*.

*P3* ‘AI could facilitate routine image evaluation and enhance efficiency, but radiographers must remain engaged in critical thinking and continuous learning to ensure patient safety and care quality.’ *(MRI and CT Radiographer with experience practising in Ghana UK)*.

#### 3.3.3 Theme 2.3: Validation and Quality Assurance

Radiographers' input is seen as irreplaceable, especially in complex or ambiguous cases where AI may misinterpret radiographic image appearances. Radiographers in this study saw AI as a tool in assuring the quality of their PCE performance rather than a standalone solution. Thus, AI may help in validating the radiographers' findings or vice versa to ensure they are accurate and represent the pathology that is being identified. A key impression is the need to ensure that a human‐centred approach to the practice of PCE is maintained.

*P7* ‘I believe that artificial intelligence will be instrumental in image interpretation, but the continued necessity of the human element in interpreting complex imaging cases remains critical, given that AI can produce reports that may misinterpret similar pathologies.’ *(CT Radiographer with experience practicing in Ghana)*.

*P9* ‘I do believe AI can assist in image interpretation but shouldn't replace radiographers, as human expertise is crucial for accurate image evaluation.’ *(CT Radiographer with experience practicing in Ghana)*.

*P8* ‘I am a specialist CT radiographer with six years of experience. I've worked mainly in the UK and spent six months in New Zealand as a CT radiographer. Our current system… works well but AI's accuracy needs verification by the radiographers.’

## 4 Discussion

This study provides insight into cross‐sectional imaging radiographers' perspectives on the ethical considerations in preliminary clinical evaluation (PCE) and the potential impact of artificial intelligence (AI) on the future sustainability of the role. The findings suggest that participants recognised the importance of ethical principles such as confidentiality, professional boundaries, and regulatory compliance when undertaking PCE. Furthermore, participants expressed cautious optimism towards AI, perceiving it primarily as a supportive tool to radiographers' decision‐making rather than a substitute for their professional judgement and expertise.

### 4.1 Ethical Considerations in PCE Practice

Participants in this study demonstrated a clear understanding of the ethical responsibilities in PCE practice, especially regarding patient confidentiality, professional accountability, and maintaining professional boundaries. These results align with general professional guidance from the SCoR, which considers confidentiality a key element of radiographic practice and ethics [43]. For example, an HCPC case involving a radiographer who shared a radiographic image containing patient‐identifiable information on social media highlights the serious professional consequences of breaching patient confidentiality, resulting in a finding of professional misconduct [44]. This case also lends credence to why ethical review of patient imaging data is very critical and must be upheld all the time. Additionally, the World Health Organisation (WHO) stresses that dignity, privacy, respect, and confidentiality should be preserved throughout patient care [45].

Beyond confidentiality, participants emphasised that ethical practice in PCE extends to the management of professional responsibility and role boundaries. While PCE is intended to support timely clinical decision‐making and enhance patient safety, it fills an important gap between image acquisition and definitive reporting of the patient's care journey, which could create potential uncertainties regarding responsibility for image interpretation and communication of findings. As such, ethical practice is not merely about adhering to regulations but also about recognising the limits of one's competence and authority. Participants' emphasis on practising within professional scope reflects an appreciation of the ethical principle of non‐maleficence, whereby practitioners seek to avoid causing harm through misinterpretation or miscommunication.

An important tension emerging from the findings relates to the balance between breaking clinically meaningful diagnostic information and avoiding the assumption of responsibilities that lie outside the radiographer's scope. Participants described situations in which patients requested information about their imaging findings. However, there was consistent agreement that preliminary clinical evaluation (PCE) findings should not be communicated directly to patients, as this falls outside the intended scope of the role. While this reflects adherence to current professional expectations, it also highlights a potential ethical dilemma in practice. In such circumstances, radiographers may experience tension between the ethical desire to support and inform patients and the requirement to remain within established professional boundaries. Although participants did not explicitly discuss such scenarios in detail, their emphasis on role boundaries and on avoiding discussion of imaging findings with patients suggests an awareness of the potential risks associated with role ambiguity. Thus, it is worth reinforcing ethical considerations and potential challenges in radiographer PCE training.

Similarly, inaccurate, poorly worded, or overconfident PCE comments could have significant consequences for patient management. The findings suggest that participants viewed cautious, appropriately framed communication as essential for minimising the risk of diagnostic error and ensuring that referrers understand the evaluation's preliminary nature. The avoidance of terms implying definitive interpretation, such as ‘confirmed’ or ‘diagnosis’, reflects an understanding that PCE is intended to support initial patient care rather than replace a formal radiological report. This distinction is important because it helps maintain transparency regarding the limits of the radiographer's contribution while reducing the risk of inappropriate clinicians' reliance on preliminary findings as a final report.

Participants also highlighted compliance with institutional policies, professional guidance, and regulatory requirements as a cornerstone of ethical PCE practice. Although adherence to such frameworks may protect practitioners from legal and professional consequences, the findings suggest that participants viewed these standards primarily as mechanisms for safeguarding patient welfare. This aligns with the WHO's policy position that ethical principles should be embedded within healthcare systems and imaging practices to protect patients from harm and promote patient‐centred care [45]. In this regard, ethical PCE practice appears to involve more than compliance with rules; it requires practitioners to continually balance patient safety, professional accountability, competence, and effective interdisciplinary communication within professional boundaries. The potential for role conflicts, miscommunication, or misinterpretation could be high; thus, the need for accountability, transparent and traceable record‐keeping, and respect for one's professional scope of practice are important ethical considerations [22, 23].

Notably, although participants demonstrated a strong understanding of confidentiality and professional boundaries, the findings also reveal an area of uncertainty. Unlike many established healthcare practices, PCE lacks a well‐developed body of literature explicitly addressing ethical decision‐making. Existing guidance focuses predominantly on governance, competence, training, and service delivery rather than the ethical complexities that may arise in practice. Consequently, radiographers may be required to navigate challenging situations using broader professional ethical principles rather than PCE‐specific ethical frameworks. This therefore calls for future research exploring ethical tensions and decision‐making processes within PCE practice, particularly as radiographers assume increasingly active roles in image interpretation.

Ethical practice is a cornerstone of healthcare provision and requires comprehensive education, ongoing professional development, and clear institutional guidance [46]. Given the expanding role of PCE in contemporary imaging services, there is a need for ethical principles to be more explicitly incorporated into PCE education, competency frameworks, and professional guidance. Such measures may better prepare radiographers to navigate the ethical uncertainties, role boundaries, and accountability considerations associated with this evolving area of clinical practice.

### 4.2 Prospects of Human‐Centred PCE in the Era of AI

The participants in this study perceived AI as a tool for augmenting, rather than replacing their role in PCE. Participants recognised the potential of AI to improve workflow efficiency, support consistency in PCE performance, and enhance quality assurance processes. These perspectives align with evidence suggesting that AI can improve diagnostic efficiency and accuracy while helping to reduce workload pressures within imaging services, particularly through image triage, flagging and prioritisation of urgent findings [27, 28, 47]. These benefits reflect the growing recognition that AI may function as a valuable decision‐support tool within radiological practice rather than an autonomous replacement for human expertise.

However, participants consistently emphasised the need for ongoing human oversight, particularly in complex, ambiguous, or clinically nuanced cases. Concerns regarding over‐reliance on AI are well documented in the literature [25, 47]. The present findings suggest that participants viewed human judgement not simply as a safeguard against technical limitations of AI but as an ethical necessity within PCE practice. PCE frequently involves contextual interpretation, clinical reasoning, and consideration of patient‐specific factors that may not be fully captured by AI algorithms. Consequently, an overdependence on AI could risk reducing critical thinking and independent clinical judgement, potentially increasing vulnerability to automation bias, whereby clinicians accept AI outputs without sufficient scrutiny.

Participants' concerns regarding the quality of training datasets and potential algorithmic biases further highlight important ethical considerations. Biases embedded within AI systems may disproportionately affect certain patient populations if the data used for algorithmic development are not sufficiently representative. In the context of PCE, this raises ethical questions surrounding fairness, equity, and justice in healthcare delivery. While AI systems may appear objective, their outputs are inherently dependent upon the quality and diversity of the data on which they are trained. Therefore, the ethical use of AI extends beyond technical performance and requires continual evaluation of whether AI‐supported decisions are equitable across different patient groups and regions where there is a lack of technological infrastructure to support the efficient implementation of AI systems.

The findings also raise important questions regarding accountability in AI‐assisted PCE practice. Participants advocated for continued radiographer oversight, suggesting an implicit recognition that responsibility for clinical decisions cannot simply be transferred to AI systems. As AI becomes increasingly integrated into radiological image interpretation workflows, uncertainties may emerge when AI outputs conflate with radiographers' professional judgement. In such circumstances, determining where responsibility lies for incorrect interpretations or subsequent patient harm becomes ethically complex. The findings suggest that maintaining radiographers as the final clinical decision‐makers is necessary not only for patient safety but also for preserving clear lines of professional accountability.

Furthermore, participants' emphasis on retaining human involvement may reflect concerns regarding the preservation of professional expertise. While AI has the potential to enhance performance, excessive dependence on AI systems could contribute to deskilling if practitioners become less engaged in active image interpretation, as previously suggested by Akudjedu et al. [25]. This presents a further ethical tension whereby technologies designed to improve patient care may inadvertently erode the very human expertise required to identify AI errors and ensure safe practice. Therefore, the challenge is not whether AI should be incorporated into PCE, but how it can be integrated in a manner that strengthens rather than diminishes professional competence. As such, it is ethically crucial that radiographers applying PCE in their daily practice maintain an adequate skill level through continuing professional development (CPD), as suggested by Smith (2006) [19].

Overall, the findings support a human‐centred approach to AI implementation in PCE, whereby AI functions as a complementary tool for decision support, second‐reading, and triage, while radiographers retain responsibility for clinical reasoning and final judgement [48, 49]. Participants' perspectives therefore extend beyond the functional benefits of AI and highlight broader ethical considerations relating to accountability, transparency, fairness, professional autonomy, and patient safety. This aligns with the Society of Radiographers' guidance, which recognises the potential value of AI in supporting image interpretation while emphasising the need for robust evidence, governance structures, and appropriate human oversight before widespread implementation in clinical practice [9].

## 5 Limitations and Future Research

Though this study provides valuable insights into radiographers' perspectives on the ethics of PCE and the radiographers' perceived impact of AI on the future of its practice, it is limited by its sample size and focus on expertise across only two imaging modalities. Additionally, the geographical scope of the sample involved was limited to those enrolled in the course; as such, the perspectives of individuals from regions not represented in the study remain unknown. This limitation might influence the transferability of the findings, particularly in contexts that differ significantly in radiography education and practice. Additionally, the study did not explore the AI tools used by participants in their clinical practice to support PCE practice. Future research could explore perspectives across other imaging modalities or include multidisciplinary viewpoints, such as those of radiographers who are actively practising, radiologists and referring clinicians on PCE ethics and AI. Also, a survey could be used to reach more participants to enhance geographical representation of views. Finally, longitudinal studies may also be beneficial in assessing how attitudes towards AI and ethical practice change over time as PCE adoption into clinical routines becomes wider, as well as the ethical tensions, dilemmas and conflicts that arise in the application of PCE in clinical practice.

## 6 Conclusion

This study has emphasised the ethical considerations important to radiographer PCE practice and their perspectives regarding the potential impact of AI on the future image interpretation practice in cross‐sectional imaging. The findings demonstrate a strong ethical awareness among participants in relation to PCE practice, especially around patient confidentiality, respect for professional boundaries, and adherence to legal and institutional frameworks. These ethical principles were considered critical for safe and responsible PCE practice.

The responsible use of AI is crucial, particularly in understanding its implications for the sustainability of the radiographers' evolving role in PCE. Regarding the implications of AI on the future role of radiographers in PCE shows optimism; however, with caution. While participants suggest that AI could enhance PCE workflow, augment radiographers' interpretation efforts and reduce workload, there is caution against complete reliance on this technology.

## Author Contributions

Messiah N.K. Anudjo: Conceptualisation, methodology, data collection, analysis and original draft preparation. Theophilus N. Akudjedu/Sharon Docherty: Conceptualisation, supervision, reviewing and editing of the original draft.

## Funding

This research did not receive any specific grant from funding agencies in the public, commercial, or not‐for‐profit sectors.

## Ethics Statement

The project received ethical approval from Bournemouth University's Research Ethics panel (Ethics Approval ID: 59938). Participants' information sheet was provided, and informed consent was obtained from each participant through a signed electronic ‘participant agreement’ form. Participants were informed of their right to withdraw from the study at any time without providing reasons.

## Consent

Written informed consent was obtained for anonymised participant information to be published in this article.

## Conflicts of Interest

The authors declare no conflicts of interest.

## Data Availability

Data required for this study may be made available by the author(s) upon reasonable request. Of note, the data to this study were drawn from part one of a PCE study exploring the perspectives of a multinational cohort of cross‐sectional imaging by the authors of this manuscript.
